# Prenatal substance exposure and maternal hostility from pregnancy to toddlerhood: Associations with temperament profiles at 16 months of age

**DOI:** 10.1017/s0954579421001000

**Published:** 2021-10-15

**Authors:** Brendan D. Ostlund, Koraly E. Pérez-Edgar, Shannon Shisler, Sarah Terrell, Stephanie Godleski, Pamela Schuetze, Rina D. Eiden

**Affiliations:** 1 Department of Psychology, The Pennsylvania State University, University Park, USA; 2 Research Institute on Addictions, University at Buffalo, State University of New York, Buffalo, USA; 3 Department of Human Development and Family Studies, The Pennsylvania State University, University Park, USA; 4 Department of Psychology, Rochester Institute of Technology, Rochester, USA; 5 Department of Psychology, State University of New York, Buffalo, USA

**Keywords:** hostility, maternal smoking in pregnancy, prenatal marijuana exposure, Research Domain Criteria (RDoC), temperament

## Abstract

We investigated whether infant temperament was predicted by level of and change in maternal hostility, a putative transdiagnostic vulnerability for psychopathology, substance use, and insensitive parenting. A sample of women (*N* = 247) who were primarily young, low-income, and had varying levels of substance use prenatally (69 nonsmokers, 81 tobacco-only smokers, and 97 tobacco and marijuana smokers) reported their hostility in the third trimester of pregnancy and at 2, 9, and 16 months postpartum, and their toddler’s temperament and behavior problems at 16 months. Maternal hostility decreased from late pregnancy to 16 months postpartum. Relative to pregnant women who did not use substances, women who used both marijuana and tobacco prenatally reported higher levels of hostility while pregnant and exhibited less change in hostility over time. Toddlers who were exposed to higher levels of prenatal maternal hostility were more likely to be classified in temperament profiles that resemble either irritability or inhibition, identified via latent profile analysis. These two profiles were each associated with more behavior problems concurrently, though differed in their association with competence. Our results underscore the utility of transdiagnostic vulnerabilities in understanding the intergenerational transmission of psychopathology risk and are discussed in regards to the Research Domain Criteria (RDoC) framework.

A mother’s mood during the transition from pregnancy to parenthood may be characterized by periods of both stability and change. Recent work in perinatology and developmental psychopathology converge on the suggestion that a mother’s emotional experience while pregnant and in the first years of life influences her child’s long-term development, including elevated risk for neurodevelopmental and behavioral problems from infancy to adolescence (see Van den Bergh et al., 2017 for review). For example, pre- and early postnatal maternal emotion marked by anxiety has been linked to infant negative affect and childhood internalizing symptoms (e.g., Lawrence, Creswell, Cooper, & Murray, 2020; Spry et al., 2020). These effects likely vary by the type, timing, and intensity of a pregnant woman’s pre- or postnatal emotional experience, which itself may be exacerbated by other stressors, such as substance use and poverty (e.g., Eiden et al., 2011). Building on these findings, we argue three significant gaps in our literature must be addressed to aid parents, practitioners, and policymakers in determining how best to support pregnant women and their infants. Specifically, studies to date have often limited the assessment of a mother’s emotional experience to monolithic clinical diagnoses, relied on static measures of maternal mental health, and have largely failed to disentangle the influence of pre- and postnatal maternal mood on infant neurobehavior.

The purpose of this study was to examine whether individual differences in infant temperament could be predicted by a mother’s level of hostility, a putative transdiagnostic vulnerability for psychopathology, substance use, and insensitive parenting (Eiden et al., 2011; Schuetze, Eiden, & Dombkowski, 2006). We assessed hostility during pregnancy and across the first 16 postnatal months in a sample of women who were primarily young, low-income, and had varying levels of substance use prenatally. Using a prospective longitudinal design, we sought to address some of the methodological limitations in the extant literature. Namely, we examined both level and change in hostility to characterize the role of pre- and postnatal maternal emotion on infant dysregulation within the context of high sociodemographic risk. In order to further knowledge on the intergenerational transmission of psychopathology risk, we integrated this approach into the National Institute of Mental Health (NIMH)’s Research Domain Criteria (RDoC) –an initiative aimed at describing patterns of both dysfunction and adaptive functioning across levels of analysis to clarify how psychopathologies manifest across development (Franklin, Jamieson, Glenn, & Nock, 2015; Insel et al., 2010; Kozak & Cuthbert, 2016).

## Hostility: a transdiagnostic vulnerability evident in the perinatal period

Perinatal psychiatric disorders are common obstetric complications linked to adverse child outcomes from birth onward (Gluckman, Hanson, Cooper, & Thornburg, 2008; Howard & Khalifeh, 2020; Van den Bergh et al., 2017). Included in these diagnostic categories, however, are heterogeneous subgroups of individuals with symptom clusters that often do not fit neatly into traditional psychiatric taxonomy (e.g., Putnam et al., 2017; Walsh et al., 2019), a common problem in clinical research. Consequently, the NIMH introduced RDoC to aid researchers in identifying intermediate phenotypes –defined by functioning along a continuum of neural, cognitive, emotional, and behavioral activity –that may explain heterogeneity within, as well as comorbidities between, psychiatric disorders (Casey, Oliveri, & Insel, 2014; Cuthbert & Insel, 2010; Insel et al., 2010). Burgeoning evidence suggests that this approach may be particularly useful when considering risk subgroups identified based on vulnerabilities that cut across diagnostic categories (Blau, Orloff, & Hormes, 2020; Lin et al., 2019; Obeysekare et al., 2020; Ostlund et al., 2019). A focus on symptoms manifested in the pre- and early postpartum period may also help identify patterns of functioning and mental health outcomes across two generations.

Transdiagnostic vulnerabilities are relatively stable, trait-like patterns of behavior that range from normal to abnormal. Exceptionally high (or low) levels of these vulnerabilities contribute to a range of psychiatric disorders, demonstrating their clinical utility (Beauchaine, Zisner, & Sauder, 2017; Dalgleish, Black, Johnston, & Bevan, 2020; Harvey, Watkins, Mansell, & Shafran, 2004; Krueger & Eaton, 2015; Nolan-Hoeksema & Watkins, 2011). However, there is a dearth of evidence describing transdiagnostic vulnerabilities among pregnant women. To address this shortcoming, Lin et al. (2019) examined whether a pregnant woman’s level of emotion dysregulation, a known contributor to psychopathology risk across the lifespan (Beauchaine, 2015; Cole, Hall, & Hajal, 2013), was associated with measures of mental health and physiological responding to stress. The authors found that a mother’s level of emotion dysregulation while pregnant was related to more chronic and episodic stress, higher trait and pregnancy-specific anxiety, more self-injurious thoughts and behaviors, and more self- and interviewer-reported depressive and borderline symptoms. They also found that pregnant women with higher levels of emotion dysregulation exhibited a blunted parasympathetic response to an ecologically valid infant cry stressor, reflecting a diminished physiological reactivity to infant cues that may thwart responsive caregiving efforts (Ablow, Marks, Feldman, & Huffman, 2013; Del Vecchio, Walter, & O’Leary, 2009; Schuetze & Zeskind, 2001). Utilizing the same sample, Ostlund et al. (2019) found that newborns whose mother reported higher levels of emotion dysregulation while pregnant exhibited blunted arousal and attention soon after birth.

Despite elevated psychopathology risk, few studies that have examined prenatal transdiagnostic vulnerabilities investigate these risk processes among pregnant women who are high risk by virtue of sociodemographic factors such as age, race/ethnicity, or socioeconomic status (e.g., Ostlund et al., 2019; see Walsh et al., 2019 for exception). Whether these transdiagnostic vulnerabilities adequately capture psychopathology risk among women who are predominately young, low-income, and used illicit substances while pregnant remains to be seen. This clustering of demographic, economic, and structural stressors increases risk for maladaptive outcomes for both a mother and her developing child, making this a particularly vulnerable population.

Here, the focus is on maternal hostility while pregnant and in the first two years postpartum, a putative transdiagnostic vulnerability related to substance use, interpersonal discord, and psychopathology. Hostility is defined by a dysfunctional pattern of cognition –one that is prone to negative, cynical, and denigrating thoughts and feelings, along with antagonistic attitudes toward and in evaluation of others (e.g., viewing others as distrustful and purposefully hurtful; Berkowitz, 1993; Buss, 1961; Miller, Smith, Turner, Guijarro, & Hallet, 1996). This oppositional cognitive bias abets intense and persistent anger and resentment, and may incite aggressive behavior (e.g., Eckhardt, Norlander, & Deffenbacher, 2004; Houston & Vavak, 1991; Ramirez & Andreu, 2006). Among women, high levels of hostility have been linked to increased rates of depression, stress, and antisocial behavior (Eiden et al., 2011; Sellers et al., 2014), as well as substance use disorders (Robinson, Brower, & Gomberg, 2001). Further, mothers who use illicit substances and are high in hostility are more likely to engage in harsh and insensitive parenting practices compared to their peers (Eiden, Peterson, & Coleman, 1999; Schuetze et al., 2006). This pattern of parenting among mothers prone to hostility has, in turn, been linked to more behavior problems in their children (Rubin, Burgess, Dwyer, & Hastings, 2003; Schuetze, Lopez, Granger, & Eiden, 2008).

## Maternal use of marijuana and tobacco while pregnant

Smokers are more likely to report negative attitudes toward other people and frequent, intense bouts of anger and aggression compared to nonsmokers (Miller et al., 1996). Trait hostility is also a consistent predictor of smoking among both men and women (e.g., Whiteman, Fowkes, Deary, & Lee, 1997) and associated with lower rates of abstinence after treatment for smoking cessation (Kahler, Strong, Niaura, & Brown, 2004). In contrast to the consistent associations between hostility and smoking, the literature on the association between hostility and marijuana use is more mixed. Studies collecting daily data on marijuana use and mood symptoms report that marijuana use is often preceded by increases in negative affect (see Wycoff, Metrik, & Trull, 2018 for review). There are also reports of acute effects of marijuana use on hostility, with some studies reporting increases in hostility following marijuana use while others have reported general decreases in negative affect, including hostility (Wycoff et al., 2018). Little is known about the associations between co-use of tobacco and marijuana and changes in hostility over time.

In one of the few studies examining hostility among pregnant smokers, authors reported that maternal hostility prospectively predicted persistent smoking during pregnancy, even in the context of other mood disorder symptoms, such as depression (Eiden et al., 2011). This is particularly important since tobacco is one of the most commonly used substances in pregnancy, with rates as high as 23%, especially among young, low-income women, as noted in the Surgeon General’s Report (U.S. Department of Health and Human Services (USDHHS), 2014). Tobacco in the form of cigarettes delivers significant amounts of chemical toxins to the fetus via the maternal bloodstream (USDHHS, 2014) and increases norepinephrine, dopamine, acetylcholine, and serotonin concentrations in the developing brain (Lichtensteiger, Ribary, Schlumpf, Odermatt, & Widmer, 1988; Slotkin et al., 2015). There are robust causal linkages between prenatal tobacco exposure and infant morbidity and mortality, including risk for prematurity, low birthweight, and sudden infant death syndrome (USDHHS, 2014). Behaviorally, prenatal cigarette exposure increases risk for irritability and higher arousal in the early neonatal period (Stroud et al., 2009), problems with self-soothing and attention/orienting in the later neonatal period (Espy et al., 2011; Stroud et al., 2016), disruptive behavior, including aggression, conduct disorder, and attention-deficit/hyperactivity disorder (ADHD) diagnosis in later childhood (Clark, Espy, & Wakschlag, 2016; Estabrook et al., 2016; USDHHS, 2014), and substance use in adolescence (Scott-Goodwin, Puerto, & Moreno, 2016; Slotkin, 2008).

Although the literature on prenatal cigarette exposure is large, there are two major open issues worth noting. First, many studies have clear methodological shortcomings that limit interpretation. These include a large number of retrospective studies that have operationalized maternal smoking in pregnancy via single item and/or self-report measures, as well as studies that lack a chemically verified, demographically similar comparison group that has abstained from smoking (Molnar et al., 2017). Perceived social stigma as well as possible legal ramifications may prevent a woman from disclosing her substance use while pregnant, making it difficult to obtain accurate information. While no approach is perfect, measuring a woman’s substance use during pregnancy via multiple methods (e.g., self-report, saliva sampling) may increase the likelihood that we are characterizing her behavior accurately and reliably. Accurately characterizing prenatal substance use is particularly important when working with a high-risk sample of pregnant women (i.e., young, single, low-income) since the prevalence of smoking in pregnancy, as well as the developmental salience of these sociodemographic risks for child outcomes, tend to be higher.

Second, many studies to date have not considered prenatal use of other substances that may have additive or synergistic effects, such as cannabis. In a special issue devoted to co-use of tobacco and cannabis in pregnancy, De Genna, Stroud, and Eiden (2019) noted that the literature on developmental outcomes is quite small despite rates of co-use ranging from 45% to approximately 85% among substance users, which are higher than the use of marijuana or tobacco alone (Chabarria et al., 2016; Coleman-Cowger, Schauer, & Peters, 2017; El Marroun et al., 2011; Ko, Farr, Tong, Creanga, &Callaghan, 2015). Marijuana use during pregnancy is concerning given significant increases in the potency of the main psychoactive component of marijuana (i.e., delta-9-tetrahydrocannabinol or THC) since the 1990s (Mehmedic et al., 2010) and the increasing perception among pregnant women that marijuana use in pregnancy is safe (Bayrampour, Zahradnik, Lisonkova, & Janssen, 2019). Both tobacco and cannabis interfere with neurotransmitter levels, brain biochemistry, and brain morphology for a developing child (Downer, Gowran, & Campbell, 2007; El Marroun et al., 2016; Scott-Goodwin et al., 2016). Results from both preclinical and human studies indicate that nicotine and tetrahydrocannabinol –the psychoactive compounds in tobacco and cannabis –interfere with dopaminergic, serotonergic, and GABAergic systems (DiNieri et al., 2011; England et al., 2017; Morena, Patel, Bains, & Hill, 2016), and alter neuronal development through their impact on nicotinic and cannabinoid receptors (see Bara, Ferland, Rompala, Szutorisz, & Hurd, 2021 and England et al., 2017; for detailed reviews). Both substances cross the placenta and enter the fetal bloodstream (Little & VanBeveren, 1996) and may have stronger additive or synergistic effects than either substance alone.

In a recent study of neonatal neurobehavior with repeated assessments across the first month of life, co-use was associated with nearly double the need for external soothing (as opposed to self-soothing) of newborns. Co-use also demonstrated 42%–75% stronger associations with lower neonatal attention and lethargy compared to tobacco exposure alone (Stroud et al., 2018). Using data from the present sample, Eiden, Schuetze, Shisler, and Huestis (2018a) found that co-use of tobacco and cannabis was also associated with higher infant autonomic dysregulation compared to tobacco alone. Finally, using data from the present sample, co-use has been associated with higher internalizing behaviors and more sleep problems among girls at preschool age (Eiden et al., 2018b) and blunted stress-reactivity patterns at school age (Eiden et al., 2020) compared to tobacco alone or nonexposed children. No studies, to our knowledge, have examined whether co-use of tobacco and marijuana impacts individual differences in temperamental reactivity, despite the fact that temperament is an early indicator of childhood psychopathology risk.

## Temperament and RDoC

Among studies that have examined pre- and early postnatal predictors of temperament, a vast majority have focused on a single dimension of infant behavior, chiefly domains of negative affect (Van den Bergh et al., 2017). Although examination of individual traits has its relative merits, this approach tends to undervalue the phenotypic heterogeneity inherent to aberrant and normative behavior that may be better captured by a constellation of traits. To this end, RDoC promotes the use of quantitative approaches that reduce complex data from multiple dimensions into homogenous phenotypes, which reflect a profile of traits related to psychological health and dysfunction (Cuthbert, 2014). This approach has rarely been applied to pediatric samples (see Karalunas et al., 2014 for an exception), limiting our understanding of the developmental trajectories of RDoC constructs and, by extension, the etiology of early childhood psychopathology.

In outlining the developmental aspect of RDoC, the NIMH recognizes that, “many areas of the child psychopathology literature (e.g., reward sensitivity, cognitive and emotional dysregulation, behavioral inhibition) serve as a more compatible model for a dimensionally based approach compared to the highly specified categories of adult psychopathology” (NIMH, 2018). Building on this definition, a young child’s behavior may be characterized as an emergent property of multiple RDoC constructs, including, but not limited to, social communication (social processes system), acute threat and frustrative nonreward (negative valence systems), attention and cognitive control (cognitive system), and motor action (sensorimotor system). These RDoC constructs map onto core dimensions of temperament that together comprise a unique constellation of proclivities that define a young child’s behavior –affect, regulation, attention, activity, and arousal (Shiner et al., 2012). We argue that temperament is well suited to serve as a link between extant developmental evidence and the RDoC framework, as temperament is a dimensionally based approach focused on the mechanisms that underlie the manifestation and change of numerous traits over time (see Ostlund, Myruski, Buss, & Pérez-Edgar, 2021 for further discussion).

While the constituent dimensions of temperament are largely agreed upon (Shiner et al., 2012), researchers still disagree on how individual differences in the constellation of a young child’s traits ought to be characterized. Recent findings point to person-centered approaches, using analytical methods such as latent profile analysis (LPA), as one promising data-driven method for identify subgroups of infants who are phenotypically similar. Underlying this approach is the assumption that the complex interplay among multiple dimensionally based temperamentrelevant behaviors gives rise to finite patterns of trait expression that are shared among subgroups of children. Classifying distinct, homogeneous groups of young children provides researchers (as well as parents) with a readily interpretable, holistic representation of multiple traits to better understand a specific child and their potential trajectory (e.g., Clauss, Avery, & Blackford, 2015; Liu et al., 2010).

Although the number of profiles tends to vary depending on sample characteristics (e.g., child’s age, assessment via parental report versus behavioral observation), most researchers identify 3–5 temperament profiles in early childhood (Beekman et al., 2015; Caspi & Silva, 1995; Gartstein et al., 2017; Janson & Mathiesen, 2008; Komsi et al., 2006; Lin, Ostlund, Conradt, Lagasse, & Lester, 2018; Planalp & Goldsmith, 2020; Putnam & Stifter, 2005; Sanson et al., 2009; Scott et al., 2016). One commonly identified profile is characterized by above-average levels of activity and negative affect. This profile is highly heritable (Planalp & Goldsmith, 2020; Scott et al., 2016), stable across early childhood (Beekman et al., 2015; Komsi et al., 2006; Van Den Akker, Deković, Prinzie, & Asscher, 2010), and predicts externalizing and, to a lesser extent, internalizing problems (Janson & Mathiesen, 2008; Sanson et al., 2009; Van Den Akker et al., 2010). Due to inconsistencies in naming conventions, this profile has been referred to by various names including *undercontrolled* (Janson & Mathiesen, 2008; Komsi et al., 2006), *high reactive* (Prokasky et al., 2017), *reactive/inhibited* (Sanson et al., 2009), and *active reactive* (Beekman et al., 2015).

A second often-identified profile is likewise characterized by high negative affect, but also includes below average levels of positive affect, activity, and regulatory capabilities (e.g., *negative reactive*, Beekman et al., 2015; *negative reactive, dysregulated*, Lin et al., 2018; unregulated, Prokasky et al., 2017; *dysregulated, negative reactive*, Scott et al., 2016). A similar profile characterized specifically by high fear relative to other dimensions of negative affect has also been identified (Beekman et al., 2015; Janson & Mathiesen, 2008; Putnam & Stifter, 2005; Van Den Akker et al., 2010). These profiles predict childhood behavior problems as well (Lin et al., 2018; Stifter, Putnam, & Jahromi, 2008), and are reminiscent of the *behavioral inhibition* typology identified by Kagan and colleagues (Garcia-Coll, Kagan, & Reznick, 1984; Kagan & Snidman, 1991).

Two additional profiles are also commonly identified. One profile includes infants who exhibit high levels of positive affect and self-regulation (e.g., *positive reactive*, Beekman et al., 2015; *high positive affect, well regulated*, Lin et al., 2018; *well regulated, positive reactive*, Scott et al., 2016), and another includes infants who are average to slightly below average levels on all temperament dimensions (e.g., *unremarkable*, Janson & Mathiesen, 2008; *moderately low reactive, moderately dysregulated*, Lin et al., 2018; *typical*, Planalp & Goldsmith, 2020; *low/low*, Putnam & Stifter, 2005; *average*, Prokasky et al., 2017). Together, these profiles offer a robust and reliable representation of infant behavior, and may serve to link a mother’s transdiagnostic vulnerabilities to psychopathology risk in her offspring.

## Present study

Our goals for the present study were threefold. First, we sought to characterize individual differences in the trajectory of maternal hostility from pregnancy to 16 months postpartum. Few studies have examined the trajectories of maternal emotionality during the transition to parenthood, with the majority of the published findings focused on clinical symptoms. Putnam and colleagues (Putnam et al., 2017), for example, found that trajectories of depressive symptoms for 95% of women in their sample (*N* = 615) were best characterized as stable or decreasing from pregnancy to 5 years postpartum. In line with these longitudinal studies of clinical symptoms, we hypothesized that, on average, hostility would decrease linearly across time, although trajectories would differ between mothers. However, to our knowledge, no study to date has examined trajectories of maternal hostility from pregnancy through the first two years postnatal. Given the dearth of research on the topic, we consider this an exploratory hypothesis that may inform future research.

Second, we examined whether level (prenatal) and change (pregnancy to 16 months postnatal) in maternal hostility were predicted by prenatal substance use. We hypothesized that pregnant women who used both marijuana and tobacco would report higher levels of hostility concurrently and would maintain higher levels over time relative to pregnant women who used only tobacco prenatally, as well as women who did not use either substance. We considered this hypothesis to be exploratory given the dearth of research on trajectories of co-use in pregnancy and maternal emotionality.

Third, we tested the hypothesis that 16-month-old infants of mothers who reported higher hostility while pregnant and maintained higher levels over time would exhibit a *dysregulated* temperament phenotype. We predicted that four temperament profiles would be identified, consistent with prior research on similarly aged infants from high-risk backgrounds (Beekman et al., 2015; Lin et al., 2018). We hypothesized that higher levels of prenatal hostility and larger increases in hostility across time would be associated with an infant’s membership in a temperament profile characterized by above average levels of negative affect and below average levels of attention and inhibitory control (e.g., *negative reactive*, Beekman et al., 2015; *negative reactive, dysregulated*, Lin et al., 2018). Consistent with prior research (Lin et al., 2018), we did not expect a mother’s substance use while pregnant to be directly related to her infant’s membership in a specific temperament profile. Given established links between prenatal substance use and maternal hostility, as well as between maternal hostility and child behavior, we explored whether maternal hostility mediated the association between a mother’s substance use while pregnant and her infant’s membership to a temperament profile.

## Method

### Procedure

All procedures for the current study were approved by the university’s Institutional Review Board (see Eiden et al., 2020 for detail discussion of sample selection). Women came in to the laboratory during each trimester of pregnancy, with informed written consent collected at the first trimester visit. Mother–infant dyads returned for additional laboratory assessments at 2, 9, and 16 months postpartum.

### Participants

Women in their first trimester of pregnancy were recruited from a local hospital at their first prenatal appointment. Women were eligible if they were < 20 weeks’ gestation, having a singleton birth, aged 18 years or older, using no illicit drugs other than cannabis, no heavy alcohol use (women drank < 4 drinks per occasion and did not average > 1 drink a day), and were able to complete the self-report screening form in English. Use of cigarettes, alcohol, and illicit substances (e.g., cannabis, methamphetamine, cocaine, opioids) were assessed via the Time Follow-Back Interview (TLFB; Sobell & Sobell, 1992) and salivary assays at the end of each trimester. Newborn meconium was also tested to determine fetal exposure to cigarettes and a range of illicit substances. Given the initial goal of the study to examine effects of maternal prenatal tobacco use on developmental mechanisms and outcomes of their offspring, tobacco users were oversampled based on this initial screener so that the closest eligible nonsmoking woman (matched on maternal age and highest educational attainment) was recruited for every two smoking women. This allowed for a full range of light to heavy smokers, and accounted for the likely scenario of greater attrition among tobacco smokers.

A total of 258 mother–infant dyads participated in the 2-month appointment, at which time they were considered officially enrolled. One mother–infant dyad was excluded from analyses because infant meconium was positive for methamphetamine, two infants were excluded because they had hydrocephaly, two were excluded because of a later diagnosis of autism, one was excluded because of maternal binge drinking during pregnancy, and one additional participant was excluded due to low maternal cognitive functioning. Finally, four participants were excluded because they were assigned to the tobacco control group, but were smoking moderate amounts of marijuana during pregnancy, resulting in a final sample size of 247 (69 nonsmokers, 81 tobacco-only smokers, and 97 tobacco and marijuana smokers).

Demographic information was collected at the first trimester appointment. Maternal age ranged from 18 to 39 (*M* = 24.09, *SD* = 5.00), and mothers were 51% African-American, 31% Caucasian, 19% Hispanic, and 8% other or mixed race with several mothers reporting more than one race. Approximately 46% percent of women were married or living with their partner at the first prenatal appointment, 33% were in a relationship but not living with their partner, 20% were single, and 1% were divorced. Approximately 29% of women had less than a high-school education, 29% completed high school, 29% completed some college courses but did not earn a degree, 9% had a vocational degree or technical training degree, and 4% received a Bachelor’s degree. Thus, the sample consisted of primarily young, unmarried, women of color with lower levels of educational attainment. Demographic information for the sample is presented in Table 1.

### Measures

#### Maternal prenatal substance use

Maternal prenatal substance use was measured through multiple methods including self-reports and biological assays. We administered the TLFB (Sobell & Sobell, 1992) toward the end of each trimester, which yielded daily data regarding maternal substance use. Mothers were provided with a calendar on which they identified approximate conception date as well as important events (e.g., holidays, birthdays, parties, sports events, anniversaries, funerals, vacations) as anchor points to aid recall. The TLFB is a reliable and valid method of obtaining daily data on patterns of substance use, including tobacco and cannabis (Robinson, Sobell, Sobell, & Leo, 2014), has good test–retest reliability, and is highly correlated with other intensive self-report substance use measures (Brown et al., 1998). The TLFB yielded data on the average number of cigarettes and joints smoked per day across the entire pregnancy, as well as the average number of standard alcoholic drinks per day across pregnancy. All mothers smoked combustible cigarettes and none were e-cigarette users.

In addition to maternal self-reports, maternal oral fluid samples were collected at each trimester and analyzed by the US Drug Testing Laboratory (Des Plaines, IL) for cotinine, the primary nicotine biomarker, and for THC, the primary psychoactive component of cannabis. Cotinine assays were conducted with enzyme-linked immunosorbent assay (ELISA) or liquid chromatography-tandem mass spectrometry (LC-MS/MS) at 10 ng/mL cutoff, and ranged from 0 to 569 ng/mL. Assays for THC were conducted with immunoassay screening (4.0 μg/L cutoff) and GC–MS confirmation (4.0 μg/L cutoff). Infant meconium samples were collected across several days after delivery until the appearance of milk stool, and were assayed with a validated LC–MSMS method (Gray et al., 2010a) at 2.5 ng/g nicotine, 1 ng/g cotinine, and 5 ng/g OHCOT, and with a validated two-dimensional GC–MS analytical method for THC, 11-hydroxyTHC; 8,11-dihydroxy-THC; 11-nor-9-carboxy-THC (THCCOOH), and cannabinol (Gray et al., 2010b). Limits of quantification for cannabinoid meconium assays were 10 ng/g for all analytes, except 11-hydroxy-THC at 15 ng/g.

Mothers were assigned to the tobacco smoking group if they self-reported smoking during pregnancy on the screener or the TLFB, if oral fluid samples were cotinine positive, or if infant meconium was positive for cotinine, nicotine, or trans-3′ hydroxycotinine (OHCOT). Mothers were assigned to the co-exposed group (tobacco and marijuana smokers) if, in addition to meeting the criteria for the tobacco group, they also self-reported cannabis use during pregnancy, or if infant meconium was positive for cannabinoids, or if maternal oral fluid was positive forΔ9-THC in any of the three trimesters. Mothers were assigned to the control group if all of the above criteria were negative each trimester during pregnancy.

#### Sociodemographic risk

Sociodemographic risk was calculated as a composite of maternal race, education, occupation, and partner status. For all items, a higher score was indicative of greater risk. The maternal race variable acted as a proxy for structural barriers and bias faced by individuals from marginalized groups. Risk was coded as positive (1) if mothers indicated that they were nonwhite (69% met this criterion). For maternal education, risk was positive (1) if the participant had not received a high school diploma or equivalent (29.1% met this criterion). Maternal occupation was coded using the Hollingshead scale (*M* = 2.06, *SD* = 1.60, Range = 1–8). The score was then divided by the maximum value of 9 in order to create a proportion, and was then recoded so that higher numbers indicated greater risk (lower occupational status). For partner status, risk was positive (1) if the participant was not married or living with a partner (54.7% met this criterion). The final sociodemographic cumulative risk variable was created by averaging the four items described above, with a possible maximum score of 1 (*M* = 0.49, *SD* = 0.25, range = 0.04–0.89).

#### Maternal pre-/postnatal hostility

Maternal hostility was self-reported in the third trimester (*M* = 2.71, *SD* = 0.72), and at child age of 2 (*M* = 2.48, *SD* = 0.71), 9 (*M* = 2.36, *SD* = 0.65), and 16 months (*M* = 2.41, *SD* = 0.70) using the Buss-Perry Aggression Questionnaire (Buss & Perry, 1992), which contains 29 items measuring anger and hostility (e.g., ‘I have become so mad that I have broken things’). Items are measured on a 5-point scale ranging from 1 (*extremely uncharacteristic of me*) to 5 (*extremely characteristic of me*), thus higher scores reflect more hostility. Internal consistency was excellent across all time points, with Cronbach’s α ranging from .91 to .92.

#### Infant temperament

Infant temperament was assessed via maternal report of the Toddler Behavior Assessment Questionnaire (TBAQ; Goldsmith, 1996) at the 16-month appointment. The TBAQ consists of 120 items measuring 11 different domains of temperament. Each item is rated on a Likert-type scale ranging from 1 (*never*) to 7 (*always*). Subscales include activity level, anger, interest, object fear, pleasure, sadness, social fear, and soothability (10 items each), as well as perceptual sensitivity (11 items), inhibitory control (13 items), and appropriate attentional allocation (16 items). Internal consistency was good for most subscales (αs ranging from .71 to .79). Reliability was lower for interest (α = .69), sadness (α = .65), social fear (α = .58), activity level (α = .58), inhibitory control (α = .54), and attention (α = .53) subscales. Descriptive information and correlations among temperament dimensions are reported in Table 2.

Infant behavioral reactivity was assessed via a standardized paradigm intended to elicit anger and frustration (Goldsmith & Rothbart, 1999). In this paradigm, infants were allowed to play with an attractive toy for 30 s while seated in a high chair. The caregiver was seated next to the child, but was asked not to engage with the child during the assessment. Once the child was engaged in the toy, a research assistant standing behind the high chair placed her hands on the child’s forearms, moved them to the child’s sides, and held them there for 30 s, while maintaining a neutral expression. After the first trial, the research assistant engaged the child with the toy for another 30 s followed by a second 30-s trial of arm restraint. The session was stopped (*N* = 5) if the child reached a maximum distress code, defined as the child reaching the highest intensity of negative affect of a full cry, or at the caregiver’s request. Following the Laboratory Temperament Assessment Battery (LabTAB) manual (Goldsmith & Rothbart, 1999), these children were assigned the maximum distress code for the remainder of the session. The child was allowed to play with the toy at the end of the two trials.

Both trials were coded in six 5-s epochs. Intensity of anger and intensity of struggle were the primary measures used in the current analyses. For each 5-s epoch, the peak intensity of anger was scored using Affex (Izard, Dougherty, & Hembree, 1983), adapted from Izard’s Maximally Discriminative Facial Movement Coding System (1979). Both peak intensity of anger and struggle were coded on a scale ranging from 0 to 3, with higher scores indicating higher intensity of anger and struggle. The affect expression scores for each 5-s epoch were averaged for each trial in order to create two composite scores for each trial, reflecting average intensity of anger and sadness for Trial 1 and Trial 2. Higher scores indicated higher arousal or reactivity. Two coders blind to all information about the families coded behavioral reactivity. Interrater reliability was calculated for 10% of the tapes with intra-class correlation coefficients ranging from .91 to .96.

#### Infant behavior problems and competences

Infant behavior problems and competence were assessed at the 16-month appointment via maternal report of the Brief Infant-Toddler Social and Emotional Assessment (BITSEA; Carter & Briggs-Gowan, 2005). The BITSEA is a 42-item scale with answers ranging from 0 (*not true/rarely*) to 2 (*very true/ often*). For the current study, the problem subscale (31 items, e.g., “Cries or has tantrums until he or she is exhausted.”) and competence subscale (11 items, e.g., “Tries to help when someone is hurt (e.g., gives a toy).”) were used. The problem subscale reflects maladaptive patterns of child behavior (e.g., age-inappropriate impulsivity, defiance, or aggressiveness), whereas the competence subscale is viewed as reflecting age-appropriate social–emotional skills, such as compliance, attention regulation, emotional awareness, and prosocial peer behavior (Carter, Briggs-Gowan, Jones, & Little, 2003). Scores on the problem scale of ≥ 15 for boys and ≥ 13 for girls indicate children in the possible problem range, while competence scores of ≤ 14 for boys and girls suggests the possible deficit/ delay range. Internal consistency of the scale was good for the problem scale (α = .83) and lower for the competence scale (α = .67).

#### Analytic strategy

Data analysis was conducted in R v4.0.2 (*r* Core Team, 2020). We first estimated latent profiles of infant temperament using standardized scores for the arm restraint and TBAQ using the *tidyLPA* package (Rosenberg, Beymer, Anderson, van Lissa, & Schmidt, 2018). Missing data were handled using *missForest* (Stekhoven & Bühlmann, 2012), an iterative machine learning imputation method (Breiman, 2001; Tang & Ishwaran, 2017). The best fitting model was determined based on the following criteria: a small Bayesian information criteria (BIC), indicating better relative model fit (Schwarz, 1978), a large entropy, indicating higher confidence in classification (Celeux & Soromenho, 1996), a minimum of 25 infants as members of the smallest profile (Lubke & Neale, 2006), and interpretable profile solutions. Infant membership to a temperamental profile from the best fitting class solution was dichotomized (yes/no) in relation to a reference group and included in subsequent analyses. We examined whether infant temperament profiles were associated with key health and demographic variables, including maternal age at recruitment, maternal relationship status, maternal education, infant sex, gestational age, birth weight, and whether the infant was born small for gestational age (i.e., age-adjusted birth weight below tenth percentile). Variables that were associated with temperament profiles were included as covariates in the path analysis (Maxwell, Delaney, & Kelley, 2018).

Next, we fit an unconditional latent growth curve model to examine level and change in maternal hostility from pregnancy to 16 months postnatal. Analyses were conducted using maximum likelihood estimation with robust standard errors (MLR) and full information maximum likelihood (FIML) in the *lavaan* package (Rosseel, 2012). Paths to observed variables were set to 1 for the latent intercept variable. For the latent slope variable, paths to observed variables were set to 0, 1, 2.75, and 4.50, to reflect the number of months from the prenatal assessment divided by a constant (4).

The unconditional latent growth curve model was then used to assess whether a mother’s substance use prenatally, as well as her hostility while pregnant and from pregnancy to 16 months postnatal, predicted individual differences in her infant’s temperament (i.e., conditional latent growth curve model). A mother’s prenatal substance use was effects coded, yielding two comparisons: (a) pregnant women who used both marijuana and tobacco or tobacco only were compared to pregnant women who did not use substances prenatally, and (b) pregnant women who used tobacco but not marijuana were compared to women who used both marijuana and tobacco. Comparison 1 examined the effect of prenatal substance use (vs. no use) on infant outcomes, while Comparison 2 assessed differences between co-use and use of tobacco alone. Model fit was considered good if the following criteria were met: a comparative fit index (CFI) > 0.95, a root mean square error of approximation (RMSEA) < 0.06, a standardized root mean square residual (SRMR) < 0.08, and a nonsignificant *χ*^2^ test (Hu & Bentler, 1999). We tested the significance of the latent growth parameters as mediators of the proposed link between prenatal maternal substance use and toddler temperament profiles within the same path model based on 10,000 bootstrapped samples with 95% bias-corrected confidence intervals (CIs). Finally, we examined post hoc comparisons between temperament profiles and the problem behavior and competences scales of the BITSEA to aid interpretation of these person-centered variables.

## Results

### Temperament profiles

**Figure 1.**
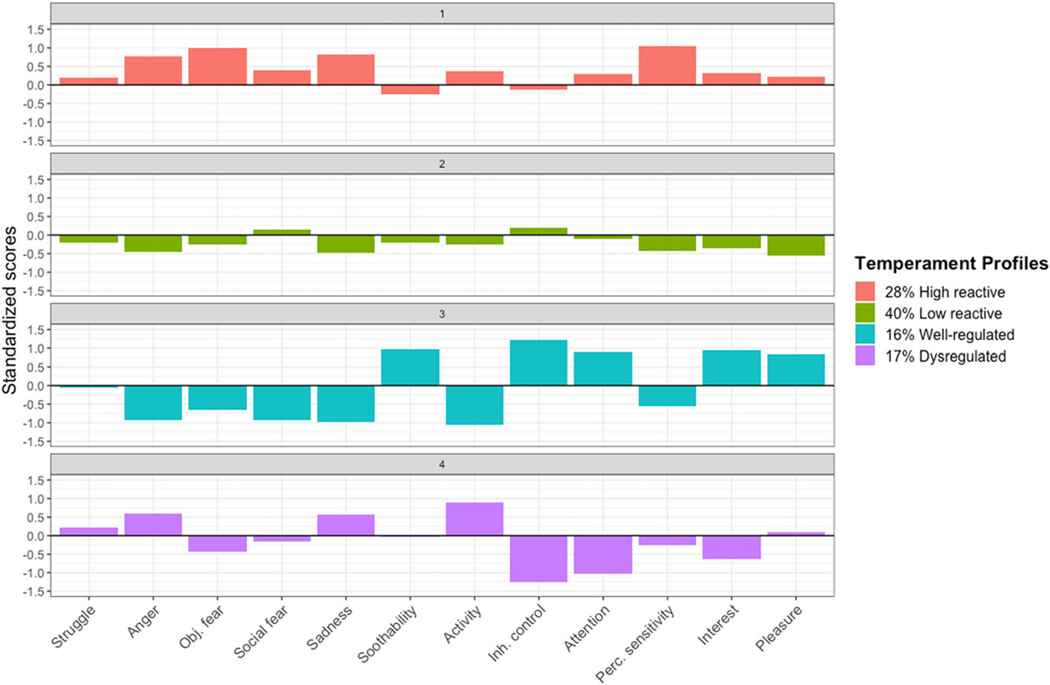
Temperament profiles at 16 months of age. Mean standardized scores on temperament dimension from the Toddler Behavior Assessment Questionnaire (Goldsmith, 1996) and an arm restraint task (Goldsmith & Rothbart, 1999) are presented. “Struggle” = behavioral reactivity to an arm restraint task at 16 months of age. “Obj. fear” = object fear. “Inh. control” = inhibitory control. “Perc. sensitivity” = perceptual sensitivity.

Descriptive data and bivariate correlations among infant temperament dimensions assessed at 16 months of age are presented in Table 2. Model fit indices, entropy, and sample size for a solution’s smallest profile are presented in Table 3. The BIC was lowest for the four-class solutions. The four- and five-class solutions had the largest entropy values. Given that it had the lowest BIC, a large entropy, and interpretable and adequately sized profiles, the four-class solution was deemed the most parsimonious. Temperament profiles from the four-class solution were used in all subsequent analyses and are presented in Figure 1 (see Supplementary Information for density plots of each temperament dimension based on profile membership).

### High reactive

The first profile consisted of 28% of the sample (*N* = 57; 27 females, 30 males). This profile was labeled *high reactive* because it comprised infants with higher levels of anger, sadness, and object fear, in combination with low levels of soothability and inhibitory control. In addition, these infants were characterized by above average perceptual sensitivity.

### Low reactive

The second profile described the plurality of infants and included 40% of the sample (*N* = 83; 38 females, 45 males). This group was named *low reactive* because these infants struggled the least in response to the arm restraint task and had above average inhibitory control. Further, these infants had moderately below average scores across the majority of domains, including interest, pleasure, anger, sadness, and perceptual sensitivity.

### Well regulated

The third profile included approximately 16% of the sample (*N* = 32; 18 females, 14 males). This group was labeled *well regulated* because these infants were characterized by low levels of negative affect –including low anger, sadness, and object and social fear –as well as high levels of inhibitory control and soothability. These infants also had lower activity levels and above average attention, interest, and pleasure. This profile was included as the reference condition in all models.

### Dysregulated

Lastly, the fourth profile comprised 17% of the sample (*N* = 35; 18 females, 17 males). This group was named *dysregulated* due to having above average levels of anger and sadness in combination with below average levels of inhibitory control. These infants were additionally characterized by high activity levels and below average attention and interest.

### Maternal hostility

**Figure 2.**
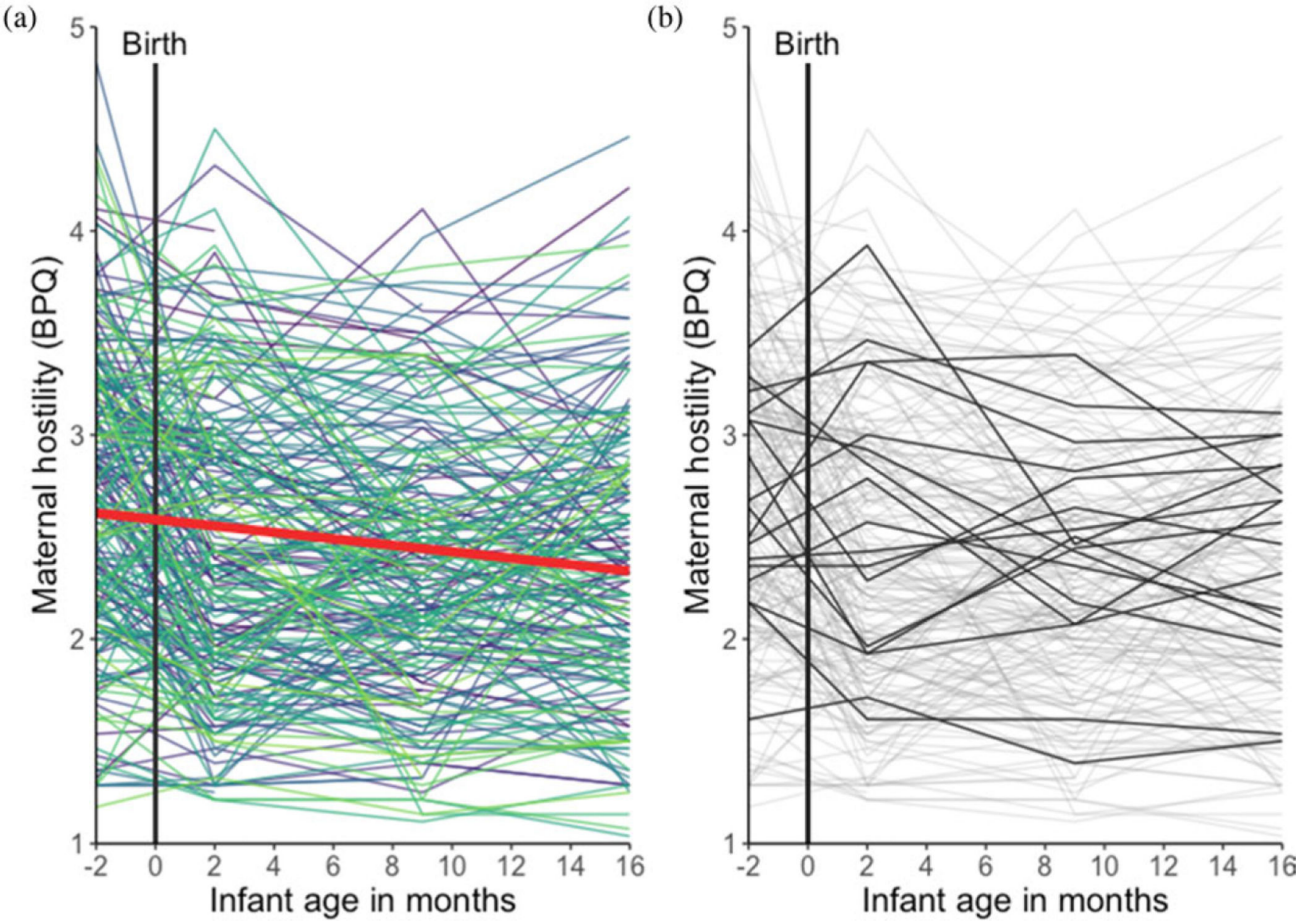
Trajectories of self-reported maternal hostility from the third trimester of pregnancy to 16 months postnatal. Data from each mother across time are represented by a unique color (***a***); the solid red line indicates linear fit. Data from a random 10% of mothers who had data at each time point are presented in black to exemplify maternal trajectories (***b***); the remainder of the sample is presented in gray.

Self-reported hostility for each mother from the third trimester of pregnancy to 16 months postnatal is presented in Figure 2.

### Repeated measures analysis of variance (ANOVA)

Results from a one-way repeated measures ANOVA showed that maternal hostility significantly differed across time points, although the effect size was small, *F*(2.88, 486.38) = 19.65, *p* < .001, $\eta_{\text{g}}^{2}$ = 0.03. Pairwise comparisons (Benjamini–Hochberg adjusted; Benjamini & Hochberg, 1995) revealed that maternal hostility while pregnant was significantly higher than hostility at each time point ( *ps* < .001). Maternal hostility at 2 months postnatal was significantly higher than hostility at 9 months postnatal ( *p* = .03), and marginally higher than hostility at 16 months postnatal ( *p* = .07). Maternal hostility at 9 and 16 months postnatal did not significantly differ ( *p* = .40).

### Latent growth curve models

The latent growth curve model adequately fit the data; *χ*^2^(5) = 32.47, *p* < .001, CFI = 0.94, RMSEA = 0.15, SRMR = 0.07. The mean level of hostility in the third trimester of pregnancy (intercept) was 2.59. The variance of the latent intercept mean = was significant (*z* = 8.61, *p* < .001), indicating that women differed in their level of hostility while pregnant. The mean slope for hostility was statistically significant and negative (*b* = −0.06, *p* < .001), indicating that, on average, maternal hostility decreased from the third trimester of pregnancy to 16 months postnatal. Trajectories of hostility did not significantly differ between mothers (*z* = 0.53; *p* = .59). The latent intercept and slope variables were not correlated (*β* = −0.05, *p* = .88).

### Path models

**Figure 3.**
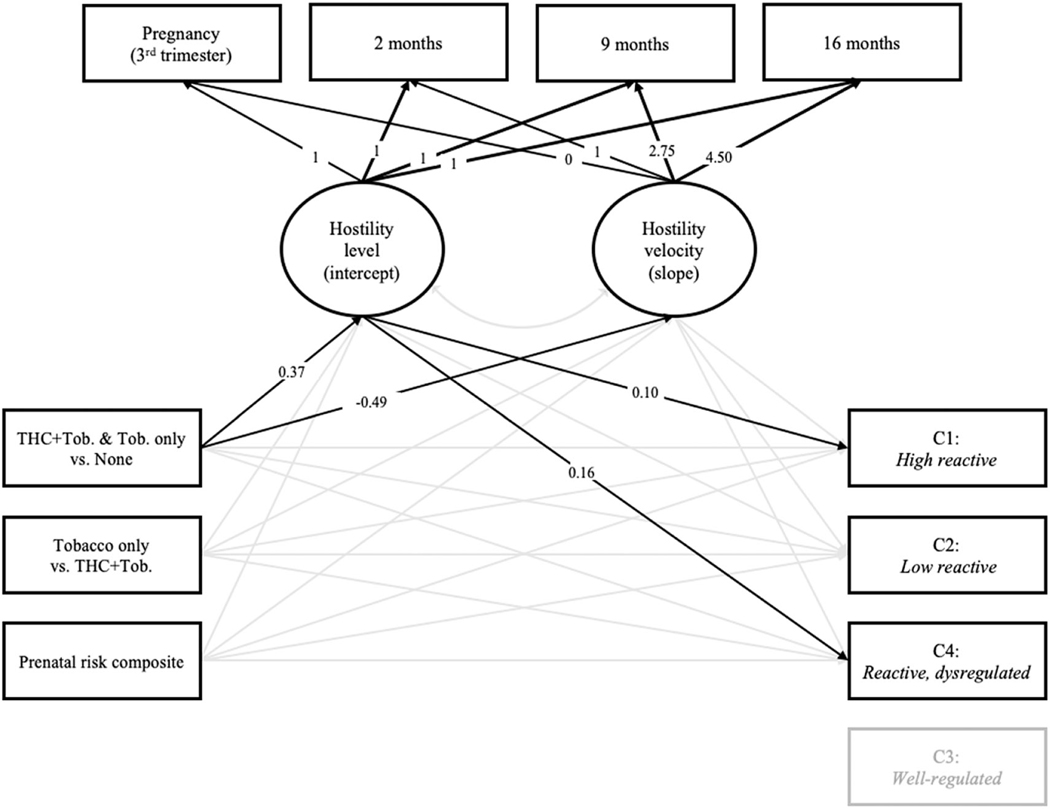
Final path model with standardized path coefficients. Solid lines reflect significant associations. A mothers’ prenatal substance use was effects coded, yielding two comparisons: (a) pregnant women who used both marijuana and tobacco or tobacco only (“THC + Tob. & Tob. only”) were compared to pregnant women who did not use either substance (“none”); and (b) pregnant women who used tobacco but not marijuana (“tobacco only”) were compared to women who used both marijuana and tobacco (“THC + Tob.”). Time coding for the latent growth variables reflects the number of months from the prenatal assessment divided by a constant (4). Temperament profiles were dummy coded; the *well-regulated* profile served as the reference group for all comparisons.

Prenatal substance use and prenatal sociodemographic risk were included as predictors of maternal and infant variables (Figure 3). The model fit the data adequately; χ^2^(20) = 61.95, *p* < .001, CFI = 0.94, RMSEA = 0.09, SRMR = 0.05. We chose the *well-regulated* profile as the reference group and excluded it from subsequent analyses based on prior findings among infants with prenatal substance exposure (Lin et al., 2018), and the fact that this profile was characterized by low negative affect and high attention, soothability, and inhibitory control (Figure 1). Associations with temperament profiles were therefore interpreted relative to the *well-regulated* profile.

Results indicate that, relative to pregnant women who did not use substances, women who used both marijuana and tobacco reported higher levels of hostility while pregnant ( *p* <.001) and exhibited less change in hostility over time ( *p* = .03). Infants of women who reported higher levels of hostility while pregnant were more likely to be classified in the *high reactive* ( *p* = .03) and *dysregulated* ( *p* = .01) profiles. No other paths were significant ( *ps* > .11). The model that included infant birth weight –the only covariate significantly associated with a temperament profile (*low reactive*) in multinomial regression analyses (all other *ps* > .19 relative to *well-regulated* reference group) –also fit the data adequately; χ^2^(22) = 54.85, *p* = .001, CFI = 0.95, RMSEA = 0.08, SRMR = 0.04. Results of this model indicate that, relative to pregnant women who did not use substances, women who used both marijuana and tobacco reported higher levels of hostility while pregnant ( *p* <.001) and exhibited less change in hostility over time ( *p* = .05). Moreover, infants of women who reported higher levels of hostility while pregnant were more likely to be classified in the *dysregulated* profile ( *p* = .04), relative to the *well-regulated* profile. No other paths were significant ( *ps* > .16). We tested whether maternal hostility while pregnant mediated the link between prenatal substance use and infant membership in either the *high reactive* or *dysregulated* temperament profiles. Neither the path to the *high reactive* profile (95% CI [−0.004, 0.026]) nor the *dysregulated* profile (95% CI [−0.005, 0.034]) was significant.

### Post hoc analyses

To establish criterion validity and aid interpretation, we conducted post hoc comparisons among the observed temperament profiles and the problem behavior (*N* = 200, *M* = 12.50, *SD* = 6.91) and competence (*N* = 201, *M* = 16.31, *SD* = 3.11) scales of the BITSEA (Carter & Briggs-Gowan, 2005). Problem behavior and competence scores were not correlated, *r*(200) = −.06, *p* = .42. One competence score from an infant in the *high reactive* profile was identified as an outlier; results remained the same when this infant was excluded from analyses (results available upon request). Problem behavior and competence scores were still not correlated, *r*(199) = −.05, *p* = .50.

**Figure 4.**
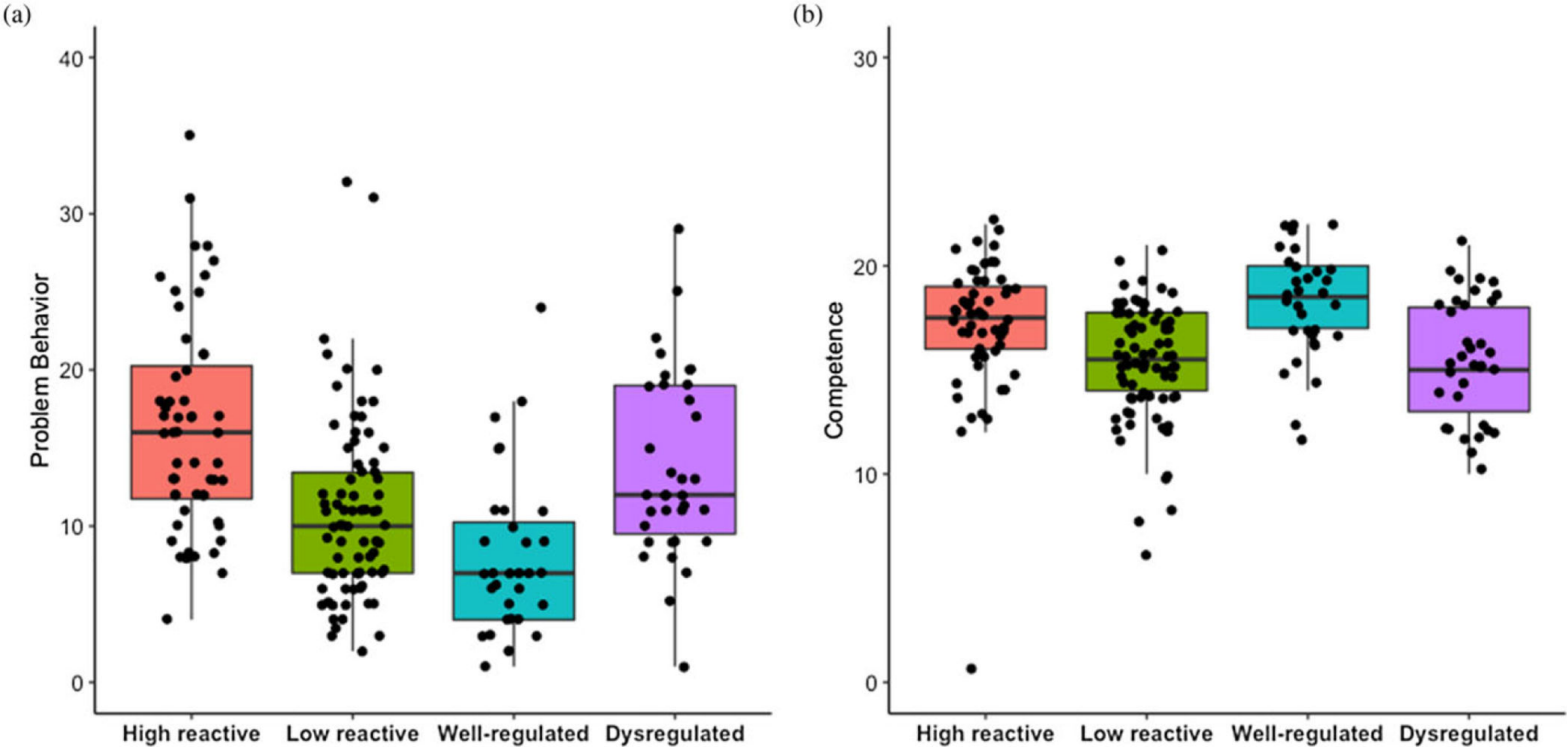
Post hoc comparisons among infant temperament profiles and the (***A***) problem behavior and (***B***) competence scales of the Brief Infant-Toddler Social and Emotional Assessment (BITSEA) (Carter & Briggs-Gowan, 2005).

Using a one-way ANOVA, we found that infant’s problem behavior significantly differed based on temperament profile membership, *F*(3,196) = 15.49, *p* < 001 (Figure 4). Tukey post hoc comparisons showed that problem behavior was higher for infants in the *high reactive* profile (*M* = 16.52, *SD* = 7.49) relative to both the *low reactive* (*M* = 10.87, *SD* = 5.78) and *well-regulated* (*M* = 7.98, *SD* = 5.23) profiles ( *ps* < .001). Problem behavior was also higher for infants in the *dysregulated* profile (*M* = 13.76, *SD* = 5.96) relative to the *well-regulated* profile ( *p* = .001), but not relative to the *low reactive* profile ( *p* = .11). Problem behavior did not differ between infants in the *low reactive* and *well-regulated* profiles ( *p* = .13), or between infants in the *dysregulated* and *high reactive* profiles ( *p* = .17).

Similarly, we found that infant’s competences significantly differed based on temperament profile membership, *F*(3,197) = 9.70, *p* < .001 (Figure 4). Tukey post hoc comparisons showed that competence was higher for infants in the *well-regulated* profile (*M* = 18.16, *SD* = 2.68) relative to the *dysregulated* (*M* = 15.51, *SD* = 2.91) and *low reactive* (*M* = 15.31, *SD* = 2.81) profiles ( *ps* < .003). Competence was also higher for infants in the *high reactive* profile (*M* = 17.14, *SD* = 3.20) relative to the *low reactive* profile ( *p* = .002) as well as the *dysregulated* profile ( *p* = .05). There was no significant difference between the *dysregulated* and *low reactive* profiles ( *p* = .99), nor between the *well-regulated* and *high reactive* profiles ( *p* = .40).

Lastly, we examined whether a mother’s level of hostility was correlated with her 16-month-old infant’s scores on the BITSEA. Infant problem behavior was positively associated with maternal hostility during pregnancy (*r* = .16, *p* = .03) and at 2- (*r* = .23, *p* = .001), 9- (*r* = .27, *p* < .001), and 16- (*r* = .27, p< .001) months postnatally. Infant competence was marginally associated with maternal hostility at 9 months postnatal (*r* = −0.13, *p* = .07); otherwise, these variables were not significantly correlated ( *p* > .21).

## Discussion

Using a prospective longitudinal design, we examined the preand postnatal influence of a transdiagnostic maternal vulnerability on infant temperament, specifically within the context of varying levels of substance use exposure in utero. Our study adds to extant evidence by showing that maternal hostility (a) decreases from the third trimester of pregnancy to 16 months postpartum, (b) positively associates with co-occurring marijuana and tobacco use while pregnant, and (c) predicts membership to two behaviorally *dysregulated* temperament profiles in their infant. This effect was specific to the prenatal period: maternal hostility while pregnant predicted specific infant temperament profiles above and beyond the influence of postnatal hostility. These temperament profiles (*high reactive* and *dysregulated*) are potential intermediate risk phenotypes on the path to childhood psychopathology. Our results underscore the utility of transdiagnostic vulnerabilities, particularly while pregnant, in understanding the intergenerational transmission of psychopathology risk.

RDoC highlights intermediate phenotypes of psychological health and dysfunction, encouraging the use of quantitative approaches that reduce data from multiple dimensions to identify individuals who are phenotypically alike. Consistent with this recommendation, we used a person-centered approach and observed four distinct, homogenous groups of infants based on twelve dimensions of temperament. These profiles mirror groups identified by multiple other researchers who have used similar analytic approaches (Beekman et al., 2015; Gartstein et al., 2017; Janson & Mathiesen, 2008; Komsi et al., 2006; Planalp & Goldsmith, 2020; Prokasky et al., 2017; Putnam & Stifter, 2005; Sanson et al., 2009; Scott et al., 2016; Van Den Akker et al., 2010), although not all studies identified the same four groups in their analyses (see Ostlund et al., 2021 for discussion).

Most notably, three of the observed temperament profiles –*high reactive*, *low reactive*, and *well regulated* –resemble groups identified in two different samples reported on by Lin et al. (2018), the only other study to our knowledge that has examined temperament profiles in infants who were prenatally exposed to substances. Pregnant women in each sample examined by Lin and colleagues had varying levels of polysubstance use, including smoking (53%–54%) and marijuana use (18%–23%), despite being recruited for either cocaine (Maternal Lifestyle Study; Lester et al., 2001) or methamphetamine (Infant Development, Environment, and Lifestyle study; Smith et al., 2006) use. The replicability of these profiles is particularly impressive given that prior studies have utilized a variety of temperament measures (e.g., Lin et al., 2018; Planalp & Goldsmith, 2020) with children of various ages (e.g., Beekman et al., 2015; Scott et al., 2016), lending support for the utility of these temperament profiles in describing phenotypically similar children beginning in the first year of life. Incorporating evidence from temperament research may serve to expand RDoC by providing an established theoretical perspective on developmental change, and should be taken into consideration going forward (Ostlund et al., 2021).

The observed profiles characterize variation on dimensions of temperament that reflect functioning along continua of multiple RDoC constructs. In our sample, activity (sensorimotor systems), anger and sadness (negative valance systems), attention, perceptual sensitivity, and inhibitory control (cognitive systems) were influential in determining an infant’s membership to a specific profile. However, when identifying phenotypes that portend childhood psychological health and dysfunction, the whole may be greater than the sum of its parts. That is, it may be the confluence of multiple RDoC constructs (characterized by temperament traits) that gives rise to individual differences in infant behavior.

For instance, given low levels of negative affect (fear, anger, sadness), high self- and co-regulatory capacities (soothability, inhibitory control, attention), high level of engagement and enjoyment in activities such as solitary play (interest, pleasure), we would expect infants in the *well-regulated* profile to show the most adaptive social and emotional outcomes. Post hoc analyses support this prediction, showing that infants in this profile are reported to have the highest levels of competence concurrently. Inhibitory control and attentional capacities modulate negative affect, become increasingly employed as a young child develops, and are used together in service of goal-directed behavior (Braungart-Rieker, Hill-Soderlund, & Karrass, 2010; Morales, Fu, & Pérez-Edgar, 2016). In this way, an infant’s behavior may be considered as an emergent property of multiple RDoC constructs that becomes increasingly intertwined across early development and may be reliably captured via person-centered temperament profiles. By measuring multiple RDoC constructs, person-centered approaches allow us to pinpoint strengths and weaknesses among homogeneous groups of infants.

It is also worth considering the caregiving environment in which an infant’s temperamental proclivities may be displayed in early childhood. Indeed, infants were more likely to be classified in the *well-regulated* profile, relative to the *high reactive* or *dysregulated* profiles, if their mothers reported lower levels of hostility while pregnant. Given the negative association between maternal levels of hostility and sensitive parenting (Eiden et al., 1999; Schuetze et al., 2006), it follows that these *well-regulated* infants may experience a more supportive, nurturing environments relative to their peers. While genetic influences between maternal hostility and infant temperament cannot be ruled out, it is worth noting that these infants may ultimately have the most adaptive outcomes by virtue of a good “match” between their dispositional traits and their parents’ proclivity toward sensitive parenting (Thomas & Chess, 1977).

Relative to the *well-regulated* profile, infants were more likely to be classified in the *high reactive* or *dysregulated* profiles if their mothers reported higher levels of hostility while pregnant. Both of these temperament profiles were related to higher levels of problem behavior concurrently. A core feature of each of these profiles is anger, suggesting homotypic continuity in the affective repertoire of a highly hostile pregnant women and her behaviorally *dysregulated* infant. Perra, Paine, and Hay (2020) identified subgroups of infants at differential risk for externalizing psychopathology at age 7 years based on early levels of angeraggressiveness (see also Liu et al., 2018). While infants in the high anger/high aggressive group tended to maintain risk over time, the authors found that warm parenting was a protective factor that protected against the escalation in aggression over early development. On the other hand, socioeconomic adversity predicted membership in the high anger/high aggressive group, while a mother’s own history of antisocial behavior predicted escalation in aggression over early development (Perra et al., 2020).

Unfortunately, the caregiving environment of mothers who are high in hostility and use marijuana and tobacco prenatally tends to be characterized by insensitivity (Eiden et al., 1999; Schuetze et al., 2006). To this end, coercion theory (Patterson, Debaryshe, & Ramsey, 1989) posits that negative reinforcement patterns in the caregiver–child relationship contribute to the development of psychopathology when paired with insensitive and inconsistent caregiving. Over time, an infant’s temperament may transact with their early caregiving environment to increase (or decrease) psychopathology risk (e.g., NICHD Early Child Care Research Network, 2004; Shaw, Lacourse, & Nagin, 2005). These temperamental differences may therefore affect the dynamic interplay between the young child and their early environment, which may lay the foundation for subsequent psychopathology risk throughout development (Crowell, Puzia, & Yaptangco, 2015).

The two behaviorally *dysregulated* temperament profiles diverge, however, when fearfulness, perceptual sensitivity, and regulatory capacities are considered. Specifically, the constellation of features that define infants in the *dysregulated* and *high reactive* profile correspond to early behavioral indices of trait irritability (Beauchaine et al., 2017; Leibenluft & Stoddard, 2015) and behavioral inhibition (Garcia-Coll et al., 1984; Kagan & Snidman, 1991), respectively. Trait impulsivity is thought to be heritable, possibly via shared sensitivity in mesolimbic dopaminergic functioning (Beauchaine et al., 2017; Gatzke-Kopp, 2011). Indeed, Field and colleagues (Field et al., 2002, 2008) noted that newborn level of peripheral dopamine is associated with maternal dopamine levels, and that dopamine levels are lower among newborns of mothers who reported high levels of anger. Elevated hostility while pregnant may further potentiate the sensitivity of mesolimbic dopaminergic functioning.

For an infant in the *high reactive* profile, the proclivity toward high perceptual sensitivity and negative reactivity may require greater co-regulatory support from their caregiver during distress recovery. The dyadic attunement needed for co-regulation is likely negatively impacted by both maternal hostility and maternal substance use, each of which are independently associated with lower levels of sensitivity (Eiden et al., 1999; Schuetze et al., 2006). Based on the prior literature (Beauchaine et al., 2017; Clauss et al., 2015), the divergence between the two profiles would suggest that infants in the *dysregulated* profile are at increased risk for irritability and externalizing problems, while *high reactive* infants are more likely to demonstrate social withdrawal and internalizing difficulties.

It is worth noting that the temperamental characteristics that define infants in the *high reactive* profile –perceptual sensitivity, attention, and negative affect –may also increase these infant’s sensitivity to both positive and negative aspects of the early caregiving environment (Ellis, Boyce, Belsky, Bakermans-Kranenburg, & van Ijzendoorn, 2011; Slagt, Semon, Deković, & van Aken, 2016). Specifically, adaptive calibration models suggest that temperamentally reactive infants show an enhanced biological sensitivity to the environment and parental behavior, which in turn is a core conduit through which the environment “gets under the skin” to shape psychobiological development (Del Giudice, Ellis, & Shirtcliff, 2011). Follow-up work will need to more closely capture the temporal dynamics of maternal hostility, maternal behavior, and infant profiles (e.g., latent transition analysis) in order to better disentangle the mechanisms influencing individual trajectories.

With this same motivation in mind, we worked to separate pre- and postnatal influences of maternal hostility on infant behavioral dysregulation by simultaneously considering level (prenatal) and change (pregnancy to 16 months postpartum). Maternal hostility during each of these periods likely operates via distinct mechanisms to affect the neural, physiological, and behavioral parameters of the young child. Prenatally, instances of acute hostility may, for example, be associated with a distinct physiological profile marked by altered sympathetic activity, which in turn may restrict uteroplacental blood flow to the fetus via catecholaminergic pathways (see Rakers et al., 2015 for animal model). Postnatally, any lasting biological shifts triggered by in utero exposure could be potentiated by the insensitive and noncontingent parenting behaviors previously associated with elevated hostility.

From pregnancy to 16 months postnatal, we observed a linear decrease in maternal hostility, a pattern that may reflect a regression to a homeostatic set point following normative biological and behavioral changes from pregnancy to postpartum (e.g., Hendrick, Altshuler, & Suri, 1998). This shift may also reflect a mother’s growing ease and confidence with the daily demands of child rearing. Thus, infants whose mothers did not display the typical decreasing trajectory may be particularly vulnerable to poor adaptive outcomes.

Of course, maternal hostility is not acting in a vacuum. Indeed, in the current sample, women who used both marijuana and tobacco reported higher levels of hostility while pregnant and exhibited less change in hostility over time. It is difficult to determine directionality when considering the association between maternal hostility and use of marijuana and/or tobacco prenatally, due to their high co-occurrence (Eiden et al., 2011). In addition, mothers in this sample were disproportionately exposed to a number of environmental and structural stressors that likely increased levels of hostility, the incidence of substance use, and when present, the differential impact of these factors on infants relative to mothers with greater structural supports (e.g., education, income, and partner status).

These broader stressors likely also impacted other known risk factors, such as sleep disturbances (e.g., Obeysekare et al., 2020; Román-Gálvez et al., 2018), relatively poor nutrition (e.g., DeCapo, Thompson, Dunn, & Sullivan, 2019; Hurley, Caulfield, Sacco, Costigan, & DiPietro, 2005), and elevated levels of stress and distress (e.g., Walsh et al.,2019). More systematic studies identifying how constellations of transdiagnostic vulnerabilities influence the development (or maintenance) of intermediate risk phenotypes in offspring may allow for more precise characterization of core dysfunctions for a mother and her infant that can be targeted by prevention efforts.

To note some additional limitations in the current study, we had multiple subscales of the TBAQ show relatively low Cronbach’s alpha values. This may be attributable, in part, to the fact that this measure was not designed with a high-risk population in mind, and may have operated differently in the current sample. We discuss this limitation further in the Supplementary Information. Nevertheless, the observed temperament profiles mirror prior work with community samples (e.g., Gartstein et al., 2017; Planalp & Goldsmith, 2020), lending support to their replicability across risk status in early childhood. An additional limitation is that mothers reported on their own and their infant’s behavior at each time point, which may inflate common-method variance. Acknowledging this potential shortcoming, we did include a behavioral observation variable (arm restraint) in the LPA. It is worth noting that behavioral reactivity to the arm restraint task had a modest influence on group differentiation (see Supplementary Figure 2). Nevertheless, recent findings suggest that a mother’s mental health may not bias her report of infant temperament (Olino, Guerra-Guzman, Hayden, & Klein, 2020), as is commonly assumed. Future research may benefit from a broader assessment of behavioral data related to temperament as a complement to parent-reported data. Furthermore, the number of infants in each of the observed temperament profiles was relatively small, even though our findings correspond to profiles observed in two other samples of infants prenatally exposed to substances (Lin et al., 2018).

It is also worth noting that the current sample of pregnant women were recruited primarily for tobacco use and a subsample also used marijuana via smoking, which may limit the generalizability of our findings among pregnant women who use marijuana only and use marijuana without smoking (e.g., edible marijuana). Given that the way in which marijuana is used may vary based on geographic location and socioeconomic status in the United States (e.g., Borodovsky, Crosier, Lee, Sargent, & Budney, 2016), future research that includes a broader definition of marijuana use and marijuana use without co-use of tobacco is warranted. Moreover, in order to include ascertainment of substance use based on multiple indices, our analyses were limited to comparing groups of women who were categorized based on prenatal cigarette and marijuana use compared to non-use. Future research might consider a recruitment strategy that is not based on a case-control design and perhaps use other methods such as propensity score matching on demographics and use the dose–response measures of substance use in analyses. This approach would complement the current findings and provide insight into whether (and how) prenatal substance use relates to early childhood temperament in a dose-dependent fashion.

A few strengths of our study are worth highlighting. We prospectively assessed a sample of primarily young, low-income women and their infants from pregnancy to 16 months postpartum. We adopted a rigorous methodology for collecting longitudinal data, which included a detailed assessment of a woman’s substance use while pregnant and the use of both parent-report and behavioral observation data to characterize infant temperament profiles. We also incorporated multiple statistical methods –latent growth curve modeling and LPA –to examine how prenatal hostility, as well as change in maternal hostility, are associated with an infant’s temperament profile membership. It is worth noting that we only examined linear change in maternal hostility over time. Yet, an infant’s development over the first 16 months of life is dramatic: a young child’s emotional, cognitive, and motor repertoire quickly evolve from rudimentary and atomized to sophisticated and contingent, introducing an ever-changing set of challenges a parent must adapt to. Future research might consider examining dynamic change (e.g., quadratic, piecewise) beginning in early pregnancy and proceeding through the first 2 years of life.

Finally, our sample of families was racially diverse (51% African-American) as well as demographically and economically high-risk (predominately young, low income, and single) relative to many studies that examine pre- and early postnatal transdiagnostic vulnerabilities. Given the underrepresentation of diverse populations in psychological research (Gatzke-Kopp, 2016), and the disparate rates of perinatal morbidity and mortality among African-American women (Moaddab et al., 2018; Saluja & Bryant, 2020; Somer, Sinkey, & Bryant, 2017), future research should build on our preliminary findings to further understand and disrupt transdiagnostic vulnerabilities for mothers and infants from historically underrepresented groups during the pre- and postnatal period.

## Conclusion

Our findings advance our understanding of the intergenerational transmission of psychopathology risk in two important ways. First, we measured trajectories of a transdiagnostic vulnerability from pregnancy to 16 months postpartum, incorporating change into our conceptualization of pre- and early postnatal maternal risk. We showed that a mother’s level of hostility while pregnant and across her infant’s early development are related to use of marijuana and tobacco prenatally and, in the case of prenatal hostility, associates with her infant’s temperament. Second, we replicated temperament profiles that have been identified by other researchers in a sample of infants with varying levels of prenatal substance exposure and psychosocial stressors, demonstrating the utility of person-centered approaches for identifying RDoC-informed phenotypes in the first two years of life. Integrating a transdiagnostic perspective into our approach to perinatal mental health and early child development may ultimately provide specific targets for preventative services aimed at reducing childhood psychopathology, via early dissemination of parenting interventions that challenge dysfunctional cognitive biases, ameliorate familial risk, and support an infant’s emotional development.

## Supplementary Material

1

## Figures and Tables

**Table 1.** Demographic information.

	*N*	*M*/%	*SD*	Range
Maternal characteristics				
Age (years)	247	24.09	5.00	18–39
Education (years)	247	12.31	1.89	7–16
African-American	126	51%		
Hispanic/Latino	47	19%		
Married/living with partner	113	46%		
Prenatal substance use				
THC & tobacco	69	28%		
Tobacco only	81	33%		
None	97	39%		
Sociodemographic risk^a^	247	0.49	0.25	0.04–0.89
Hostility (BPQ)^b^				
Prenatal	229	2.71	0.72	1.18–4.82
2 months	241	2.48	0.71	1.21–4.50
9 months	210	2.36	0.65	1.11–4.11
16 months	200	2.41	0.70	1.04–4.46
Infant characteristics				
Female	116	47%		
Gestational age (weeks)	247	38.89	1.82	26–42
Birth weight (grams)	247	3234.09	577.57	767–4795
Birth length	241	50.10	2.78	33.00–57.50
Small for gestational age	31	12.65%		

a Sociodemographic risk composite of maternal race, education, occupation, and partner status.

b Buss–Perry Aggression Scale (Buss & Perry, 1992).

**Table 2:** Descriptive information and correlations among temperament dimensions.

	1.	2.	3.	4.	5.	6.	7.	8.	9.	10.	11.	12.
1. Struggle	—											
2. Anger	0.12	—										
3. Object fear	−0.01	0.44***	—									
4. Social fear	−0.14	0.21**	0.25***	—								
5. Sadness	0.19*	0.65***	0.38***	0.19*	—							
6. Soothability	0.13	−0.29***	−0.24***	−0.21**	−0.17*	—						
7. Activity	0.14	0.43***	0.13	0.16*	0.44***	−0.17*	—					
8. Inhibitory control	−0.11	−0.51***	−0.08	−0.15*	−0.35***	0.31***	−0.51***	—				
9. Attention	0.06	−0.15*	0.06	−0.07	−0.14	0.22**	−0.30***	0.48***	—			
10. Perceptual sensitivity	0.09	0.44***	0.58***	0.23**	0.42***	−0.04	0.27***	−0.14*	0.12	—		
11. Interest	0.12	−0.09	0.15*	−0.08	−0.04	0.21**	−0.20**	0.31***	0.61***	0.23**	—	
12. Pleasure	0.15*	0.09	−0.04	−0.28***	0.13	0.46***	0.06	0.06	0.16*	0.17*	0.36***	—
*N*	190	199	194	197	189	199	200	194	196	198	198	200
Mean	1.47	3.92	2.50	3.93	3.60	5.09	4.35	3.84	3.93	3.35	4.06	5.48
*SD*	0.99	1.12	1.00	0.95	0.94	0.88	0.84	0.71	0.60	0.98	0.95	0.88

Note:

* *p* < .05

** *p* < .01

*** *p* < .001.

“Struggle” = behavioral reactivity based on observational assessment of an arm restraint task (Goldsmith & Rothbart, 1999). Scores on all temperament dimensions excluding “Struggle” were calculated based on maternal report of infant behavior at 16-months of age via the Toddler Behavior Assessment Questionnaire (TBAQ; Goldsmith, 1996). Scores for TBAQ subscales were not calculated if subscale was missing 50% of their respective items.

**Table 3.** Summary of fit statistics for latent class analysis of infant temperament.

Class	BIC	Entropy	Smallest profile
1	7165.24	—	—
2	6940.50	0.77	84 (41%)
3	6880.06	0.80	32 (16%)
**4**	**6819.74**	**0.82**	**32 (16%)**
5	6841.12	0.82	23 (11%)

*Note: N* = 207.

BIC = Bayesian information criteria
