# Supplementary material for: Prenatal substance exposure and maternal hostility from pregnancy to toddlerhood: Associations with temperament profiles at 16 months of age
Source: Dev Psychopathol. Author manuscript; Available in PMC 2022 Jun 1. (PMC8794013; doi:10.1017/s0954579421001000)

**Supplementary Information**

**I.** Reanalyzing Cronbach’s alpha and group membership with problematic TBAQ subscales items removed

**II.** Density plots of temperament dimensions included in the latent profile analysis

**I.** Reanalyzing Cronbach’s alpha for TBAQ subscales with problematic items removed

As reported in the main text, multiple subscales of the TBAQ show relatively low Cronbach’s alpha values. Posthoc analyses were conducted to determine if removal of problematic TBAQ items might improve reliability. Problematic items were identified based on poor factor loadings (< .20) in a confirmatory factor analysis; sixteen (out of 120) items were excluded based on this criterion. Internal consistencies changed minimally with problematic items excluded. Reliability for each subscale was as follows: activity level (α = .60), anger (α =.81), attention (α =.49), inhibitory control (α = .51), interest (α = .76), object fear (α = .79), perceptual sensitivity (α = .72), pleasure (α = .78), sadness (α = .73), social fear (α = .53), and soothability (α = .71). We conducted a latent profile analysis using newly calculated subscales that did not include problematic items. Model fit indices, entropy, and sample size for a solution’s smallest profile are presented in Supplementary Table 1. The BIC was lowest for the five-class solutions. The five-class solutions also had the largest entropy values. However, membership in one profile from the five-class solution was exceptional small (*n* = 13). Given that it had the second lowest BIC, the second largest entropy, and interpretable and adequately-sized profiles, the four-class solution was deemed the most parsimonious. Temperament profiles from the four-class solution were nearly identical the profiles observed in the primary analyses and are presented in Supplementary Figure 1.

**Supplementary Table 1.** Summary of fit statistics for latent class analysis of infant temperament.

| **Class** | **BIC** | **Entropy** | **Smallest profile** |
| --- | --- | --- | --- |
| 1 | 7165.24 | — | — |
| 2 | 6902.93 | 0.82 | 102 (49%) |
| 3 | 6851.10 | 0.80 | 30 (15%) |
| 4 | 6788.80 | 0.83 | 31 (15%) |
| 5 | 6727.35 | 0.89 | 13 (6%) |

*Note:* *N* = 207

**Supplementary Figure 1.** Temperament profiles at 16-months of age. Mean standardized scores on temperament dimension from the Toddler Behavior Assessment Questionnaire (Goldsmith, 1996) and an arm restraint task (Goldsmith & Rothbart, 1996) are presented. “Struggle” = behavioral reactivity to an arm restraint task at 16-months of age. “Obj. fear” = object fear. “Inh. control” = inhibitory control. “Perc. sensitivity” = perceptual sensitivity.


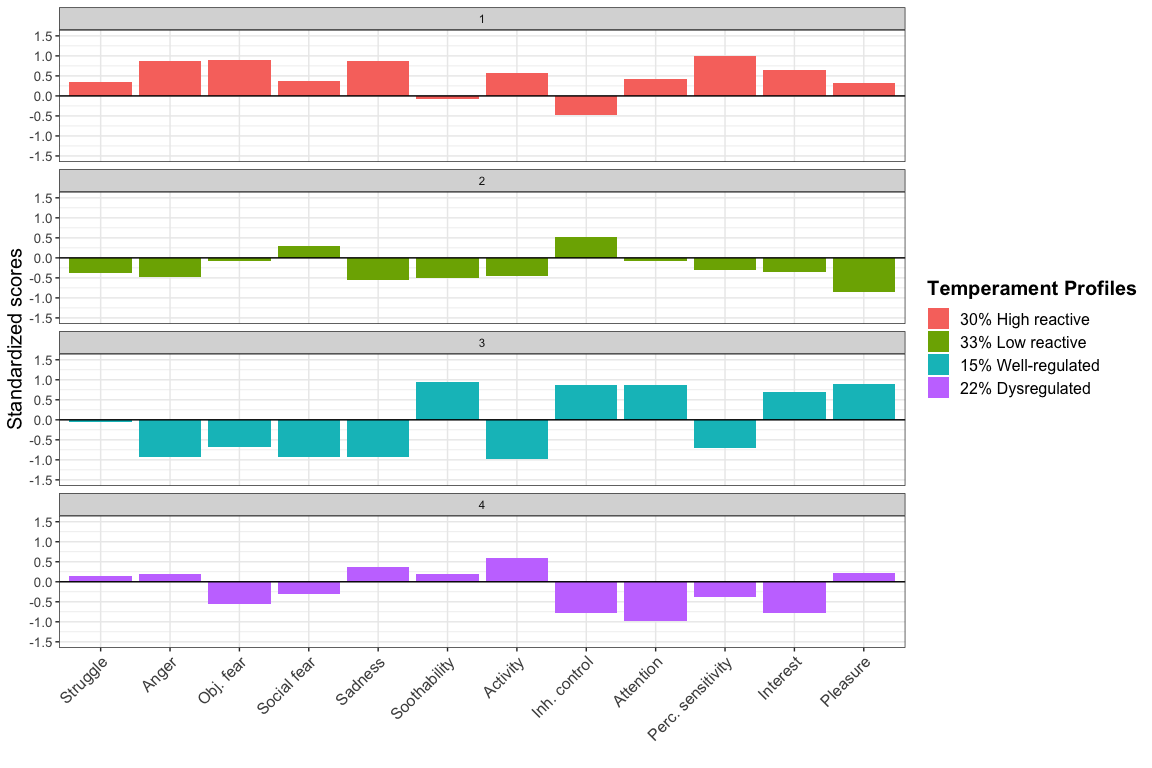


**II. Supplementary Figure 2.** Density plots of temperament dimensions included in the latent profile analysis based on profile membership.


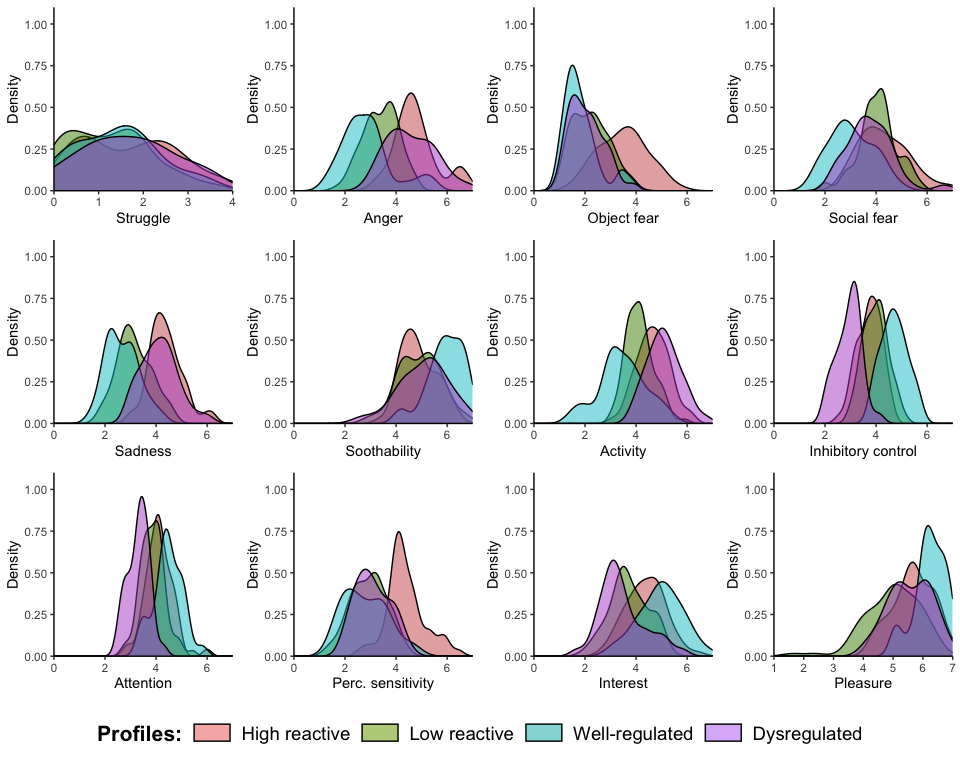

Supplement: 1 [file NIHMS1725621-supplement-1.docx]
